# Supplementary material for: Conditional chemoconnectomics (cCCTomics) as a strategy for efficient and conditional targeting of chemical transmission
Source: eLife. 2024 Apr 30;12:RP91927. doi: 10.7554/eLife.91927 (PMC11060718; doi:10.7554/eLife.91927)
Supplement: Figure 3—source data 3. [file elife-91927-fig3-data3.zip › Figure 3 source data3 part 2.zip!/Figure 3-Source data 3-part2/1002-9-CRISPResso_Report_e53dd8m5.zip!/CRISPResso_on_1002_11/9.Alleles_frequency_table_around_sgRNA_gtatctgtccagctacgtga.pdf]

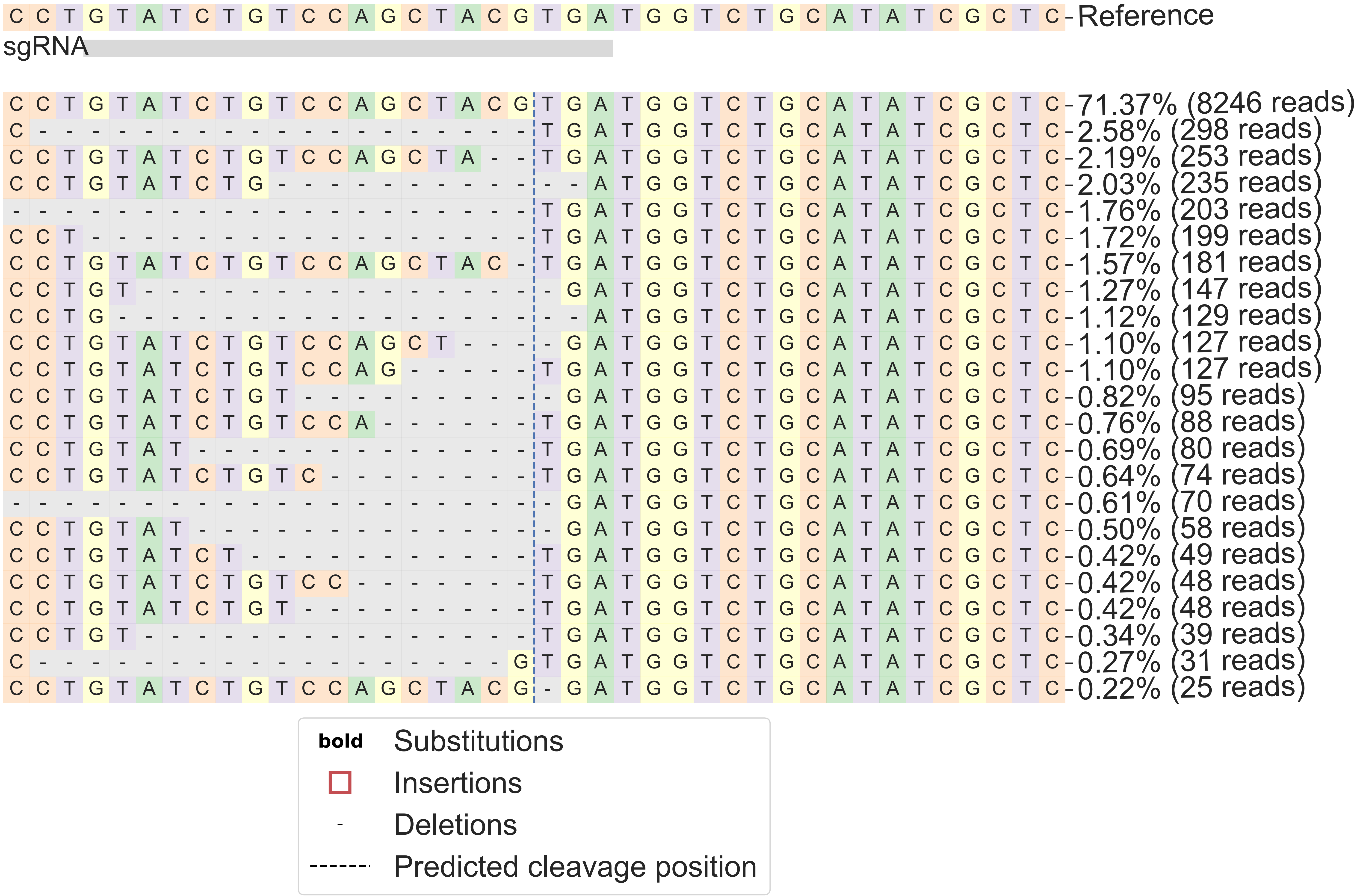
CCTGTATCTGTCCAGCTACGTGATGGTCTGCATATCGCTC - Reference
sgRNA
CCTGTATCTGTCCAGCTACGTGATGGTCTGCATATCGCTC - 71.37% (8246 reads)
C-------------------TGATGGTCTGCATATCGCTC - 2.58% (298 reads)
CCTGTATCTGTCCAGCTA--TGATGGTCTGCATATCGCTC - 2.19% (253 reads)
CCTGTATCTG------------ATGGTCTGCATATCGCTC - 2.03% (235 reads)
--------------------TGATGGTCTGCATATCGCTC - 1.76% (203 reads)
CCT-----------------TGATGGTCTGCATATCGCTC - 1.72% (199 reads)
CCTGTATCTGTCCAGCTAC-TGATGGTCTGCATATCGCTC - 1.57% (181 reads)
CCTGT----------------GATGGTCTGCATATCGCTC - 1.27% (147 reads)
CCTG------------------ATGGTCTGCATATCGCTC - 1.12% (129 reads)
CCTGTATCTGTCCAGCT----GATGGTCTGCATATCGCTC - 1.10% (127 reads)
CCTGTATCTGTCCAG-----TGATGGTCTGCATATCGCTC - 1.10% (127 reads)
CCTGTATCTGT----------GATGGTCTGCATATCGCTC - 0.82% (95 reads)
CCTGTATCTGTCCA------TGATGGTCTGCATATCGCTC - 0.76% (88 reads)
CCTGTAT-------------TGATGGTCTGCATATCGCTC - 0.69% (80 reads)
CCTGTATCTGTC--------TGATGGTCTGCATATCGCTC - 0.64% (74 reads)
---------------------GATGGTCTGCATATCGCTC - 0.61% (70 reads)
CCTGTAT--------------GATGGTCTGCATATCGCTC - 0.50% (58 reads)
CCTGTATCT-----------TGATGGTCTGCATATCGCTC - 0.42% (49 reads)
CCTGTATCTGTCC-------TGATGGTCTGCATATCGCTC - 0.42% (48 reads)
CCTGTATCTGT---------TGATGGTCTGCATATCGCTC - 0.42% (48 reads)
CCTGT---------------TGATGGTCTGCATATCGCTC - 0.34% (39 reads)
C------------------GTGATGGTCTGCATATCGCTC - 0.27% (31 reads)
CCTGTATCTGTCCAGCTACG-GATGGTCTGCATATCGCTC - 0.22% (25 reads)
bold Substitutions
Insertions
Deletions
Predicted cleavage position
